# Polymer Optical Fiber Bragg Gratings in CYTOP Fibers for Angle Measurement with Dynamic Compensation

**DOI:** 10.3390/polym10060674

**Published:** 2018-06-17

**Authors:** Arnaldo Leal-Junior, Antreas Theodosiou, Camilo Díaz, Carlos Marques, Maria José Pontes, Kyriacos Kalli, Anselmo Frizera-Neto

**Affiliations:** 1 Graduate Program of Electrical Engineering of Federal University of Espirito Santo, 29075-910 Vitória, Brazil; c.rodrigruez.2016@ieee.org (C.D.); mjpontes@ele.ufes.br (M.J.P.); frizera@ieee.org (A.F.-N.); 2 Photonics and Optical Sensing Research Laboratory, Cyprus University of Technology, Limassol 3036, Cyprus; theodosiou.antreas@gmail.com (A.T.); kkalli@cytanet.com.cy (K.K.); 3 Instituto de Telecomunicações, Campus Universitário de Santiago, 3810-193 Aveiro, Portugal; carlos.marques@ua.pt

**Keywords:** polymer optical fiber, fiber Bragg gratings, CYTOP, curvature sensor, hysteresis

## Abstract

This paper demonstrates the use of polymer optical fiber Bragg gratings (POFBGs) for angle measurements over a range of different oscillatory frequencies. The POFBGs are inscribed in low-loss, cyclic transparent amorphous fluoropolymers (CYTOP) and are imprinted using the direct-write, plane-by-plane femtosecond laser inscription method. As the polymer has a viscoelastic response and given that the Young’s modulus depends on the oscillatory frequency, a compensation technique for sensor frequency cross-sensitivity and hysteresis is proposed and verified. Results show that the proposed compensation technique is able to provide a root mean squared error (RMSE) reduction of 44%, and a RMSE as low as 2.20° was obtained when compared with a reference potentiometer. The hysteresis reduction provided by the proposed technique is 55%, with hysteresis <0.01. The results presented in this paper can pave the way for movement analysis with POFBG providing higher sensitivity and low hysteresis over a large range of motion frequencies.

## 1. Introduction

Polymer optical fibers (POFs) present advantages related to their material features. These advantages include higher fracture toughness; strain limits, and stress sensitivity due to the lower Young’s modulus and flexibility in bending when compared with silica optical fibers. For these reasons, POFs have been applied to measure parameters such as strain [1], liquid level [2], pressure [3,4], temperature [5], acceleration [6] and humidity [7]. In addition, the higher fracture toughness and strain limits enable POFs to be used as curvature sensors [8].

In motion analysis, curvature sensors are of great importance, since this analysis is generally made with respect to joint angle measurement [9]. There are electro-mechanical technologies that are typically used for this application, such as electro-goniometers, inertial measurement units (IMUs) and encoders. However, electro-goniometers and encoders need to be carefully positioned in the system under test, and may inhibit the natural pattern of motion; they are also sensitive to misalignments [10]. Furthermore, IMUs need frequent calibration and, as also happens with electro-goniometers and encoders, such sensors are sensitive to electromagnetic interference [10].

In order to overcome some of the issues presented by the aforementioned sensors, POF sensors have been applied as a wearable solution for knee angle measurement [11]. In addition, POF curvature sensors are applied in gait assistance devices such as active orthosis [12], actuators [13] and exoskeletons [14]. The POF sensors are based on intensity variation. Although this offers a low-cost technology, it does not have the multiplexing capabilities that fiber Bragg gratings (FBGs) can offer, which is important for movement analysis that generally occurs in three-dimensional planes. In addition, for a broader analysis, additional measurement points are needed, which can be accomplished in the same fiber when the FBG sensing technology is used [15].

There have been substantial and recent developments in the fabrication of polymer optical fiber Bragg gratings (POFBGs) [16,17], with gratings inscription times as low as 15 ns [18], fast inscription in different POF materials [19] and femtosecond (*fs*) laser inscription in doped POFs [20]. However, these inscription methods were applied in microstructured POFs (mPOFs), which, to date, are not widely commercially available, where only a few companies commercialize those fibers. Additionally, mPOFs have some disadvantages, such as microstructure degradation as impurities enter the holes, light scattering at the hole walls and connectorization difficulties resulting from material and numerical aperture mismatch with conventional fibers [21].

Commercially available POFs have larger core diameters when compared with mPOFs and multimode operation, which leads to POFBGs with multi-wavelength-peak, reflection spectra [22]. In order to limit the adverse effects of this multi-peak behavior, the grating inscription parameters are carefully optimized using fs laser inscription using the plane-by-plane inscription method presented in [23,24], where a single reflection peak was obtained through the control of the core mode excitation. This was demonstrated in cyclic transparent amorphous fluoropolymers (CYTOPs), which are commercial fibers that have low optical losses in the C-band, making it possible to combine POFBG sensors with off-the-shelf components and commercial FBG interrogators [25].

In addition to the higher optical attenuation when compared with silica optical fibers [26], another disadvantage of POFs is related to their viscoelastic nature, which leads to a non-uniform response under mechanical loading, such as strain, stress and force [27]. The POF creep and recovery responses were characterized in [1,28]. Moreover, the Young’s modulus variation with temperature and strain cycle frequency is characterized for polymethyl methacrylate (PMMA) and cyclic olefin copolymer (COC) POFs in [29,30]. With reference to curvature sensors and applications for movement analysis, it is anticipated that the POF sensor will have a cross-sensitivity with respect to the movement frequency that results from its Young’s modulus variation. Moreover, the flexion/extension cycle frequencies also have a direct relation with sensor hysteresis, as demonstrated and compensated in [8].

In order to combine the advantages of POFs and FBGs, in this paper, a POFBG curvature sensor is developed with CYTOP fibers, which are commercially available and present lower optical losses at C-band than other POFs. Since the material behavior can lead to hysteresis and cross-sensitivity with the movement frequency, the Young’s modulus variation with respect to strain cycle frequency is characterized using dynamic mechanical analysis (DMA). Following the material characterization, a compensation technique to mitigate the effects of hysteresis and frequency cross-sensitivity is proposed and verified for different movement frequencies of the optical sensor. The technique is based on the experimental characterization of the sensor in different frequencies, similar to the one presented in [8] for intensity variation-based sensors. However, the technique proposed in [8] does not account for the frequency cross-sensitivity in the sensor’s response and the technique proposed here can be regarded as an important improvement of the previously presented technique. Potential applications of the developed sensor include angle measurements in a robotic exoskeleton [12], as a wearable sensor for joint angle measurements [14] and on the instrumentation of robotic actuators [13].

## 2. Theoretical Background and Compensation Technique 

Equation (1) shows the well-known expression for the wavelength shift of FBGs with respect to temperature and strain:

(1) $$\Delta \lambda_{B}=\left[(1-P_{e})\varepsilon +(\alpha +\xi )\Delta T\right]\lambda_{B},$$

where *P_e_* is the photo-elastic constant, *ε* is the strain, Δ*T* is the temperature variation, *λ_B_* is the Bragg wavelength, *α* is the material thermal expansion coefficient and *ξ* is the thermo-optic coefficient. The tests are performed with constant temperature conditions, hence Δ*T* is zero and the wavelength shift presented in Equation (1) depends only on the strain applied to the fiber. The fiber curvature leads to a strain variation, which results in the wavelength shift and the direct relation between the wavelength shift and the fiber curvature is obtained as will be presented in the following. 

If the POF is under curvature, the strain in the normal direction (parallel to the fiber length) is defined as [31]:

(2) $$\varepsilon =\frac{1-\upsilon^{2}}{E(f)}\sigma ,$$

where *E* is the material Young’s modulus, *υ* is the Poisson’s ratio and *σ* is the stress, which is defined in Equation (3) for the bending case [31].

(3) $$\sigma =-\frac{Mx}{I}.$$

Regarding Equation (3), *M* is a bending constant, *x* is the displacement in vertical direction, which is caused by the fiber curvature and *I* is the cross-section moment of inertia. Thus, considering the constant temperature and the dependency of the Young’s modulus with the oscillatory movement frequency (*f*), which will be characterized in the next sections, Equations (1)–(3) can be rewritten as:

(4) $$\Delta \lambda_{B}=\left[-(1-P_{e})\frac{1-\upsilon^{2}}{E(f)}\frac{Mx}{I}\right]\lambda_{B}.$$

**Figure 1:**
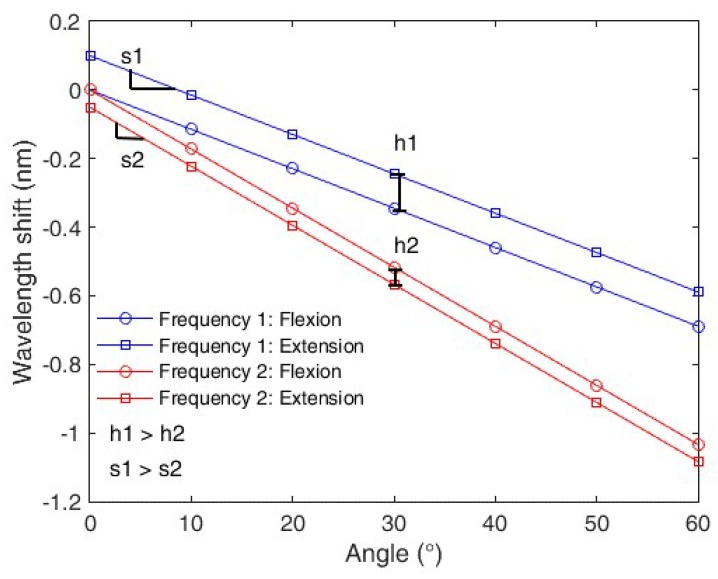
Schematic illustration of the frequency effect on the sensor sensitivity and linearity for two different frequencies; where *h*1 and *h*2 are the hysteresis for the frequencies 1 and 2, respectively. Analogously, *s*1 and *s*2 are the sensitivities for frequencies 1 and 2, respectively.

The expression presented in Equation (4) shows the sensor sensitivity with respect to the curvature (represented by *x*). As can be seen in Equation (4), the Young’s modulus variation leads to a variation in the sensor curvature sensitivity. Additionally, the Young’s modulus presents a linear variation with respect to the frequency, which leads to a sensitivity reduction of the POFBG sensor when the frequency increases. Such a Young’s modulus variation is characterized as a function of the movement frequency in Section 4. In addition, the polymer viscoelastic response also can show a dependency with the applied strain rate, which explains the relation between hysteresis and angular velocity reported in [8]. Thus, the angular velocity and, consequently, the frequency can influence both the sensor sensitivity and hysteresis, which is defined as the offset between the loading and unloading curves. The reason for this frequency dependency is related to the polymer relaxation, where in low frequency the polymer will present a higher degree of relaxation that results in higher sensitivity and hysteresis. In the curvature case, the loading curve will be referred to as the flexion cycle, whereas the unloading as the extension cycle. Figure 1 summarizes the effect of the oscillatory movement frequency on the sensor’s response. It is worth noting that Figure 1 illustrates the frequency effects that will be experimentally confirmed in the next sections. The sensitivity is the slope of the curve relating wavelength shift and angle. Thus, the frequency influence on the sensor sensitivity is represented by a slope variation; whereas the hysteresis is shown as an offset for the measurements made at the two different frequencies, where the hysteresis is defined as the difference between the flexion and extension cycles of the sensor [12]. Referring to Figure 1, *s*1 and *s*2 are the sensitivities for frequency 1 (f1) and 2 (f2), respectively. Additionally, *h*1 is the hysteresis at f1, whereas *h*2 is the one at f2. In the case presented in Figure 1, frequency 2 is higher than frequency 1. The simulated responses presented in Figure 1 are obtained through the application of Equation (4) for two different frequencies (f1 and f2) using the simulation parameters presented in Table 1, where the parameter *x* is the angular variation from 0° to 60°. In order to account for the hysteresis effects, two different offsets are applied in each curve (for f1 and f2). It is worth to mention that we estimate two offsets and two Young’s modulus, one for each frequency in order to obtain the hysteresis and sensitivity variation in the simulated responses for both cases and the actual values of the Young’s modulus and hysteresis are experimentally obtained in Section 4 through the DMA and curvature tests (at different frequencies), respectively.

**Figure 2:**
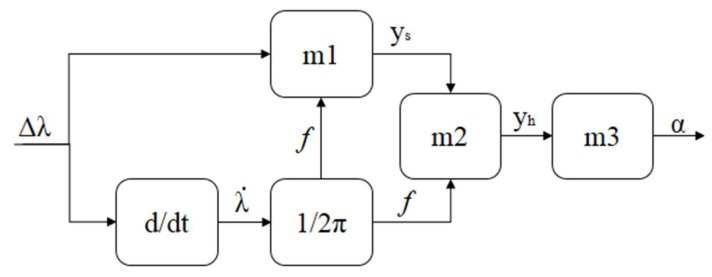
Block diagram of the sensitivity compensation (block m1), hysteresis compensation (block m2) and the correlation equation that results in the angle output (block m3).

In order to compensate the effects illustrated in Figure 1, the movement frequency needs to be estimated. Since the frequency presents a direct relation with the angular velocity, the movement frequency can be estimated by taking the angle variation rate of the POFBG curvature sensor, which can be estimated by taking the derivative of the sensor response (see Figure 2), as it is only due to the angle variation in this case. In addition, the Young’s modulus with respect to frequency requires characterization and the curvature sensor is tested under different velocities to obtain the relation between the sensor sensitivity and the movement frequency. The hysteresis between flexion and extension cycles is subsequently analyzed at each discrete frequency, which results in an experimental model relating the frequency and hysteresis. This model is applied to the POFBG curvature sensor as the hysteresis compensation. Hence, there are two models: One for the cross-sensitivity mitigation and the other for the hysteresis compensation. Figure 2 presents the block diagram of the proposed compensation technique, where the model input is the wavelength shift and the output is the compensated angle.

Regarding Figure 2, the block “m1” refers to the sensitivity compensation with respect to frequency, whereas the block “m2” is the hysteresis compensation model. The outputs of models “m1” and “m2” are y_s_ and y_h_, respectively. Both models are obtained through the experimental characterization performed in the next sections. Finally, the model “m3” correlates the compensated wavelength shift with the angle (α) that results in the compensated angle measurement. In addition, y_s_ is the sensor response with the frequency cross-sensitivity compensation, which is obtained through a linear regression of the sensor sensitivities as a function of the frequency. After obtaining y_s_, the hysteresis at each frequency is analyzed and an exponential regression is obtained, where y_h_ is the sensor responses for flexion and extension cycles obtained after the hysteresis compensation. Thereafter, a linear regression between the POFBG wavelength shift and the angle is made, where the compensated angle (α) is obtained. The proposed technique is based on the sensor characterization at different frequencies. For this reason, it accounts not only the sensor hysteresis, but also the creep or stress relaxation typically found in viscoelastic materials [27], since the viscoelastic effects occur simultaneously when the POF is under stress or strain.

## 3. Experimental Setup

**Figure 3:**
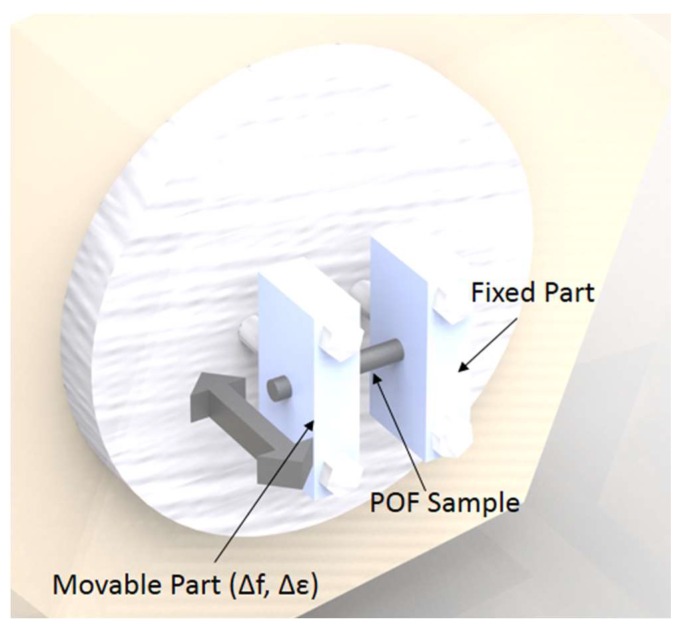
Polymer optical fiber (POF) fixation in the dynamic mechanical analysis (DMA) used in the Young’s modulus characterization with respect to the frequency.

We provide some details of the POF that is employed in the experiments. The fiber is a gradient index CYTOP (Chromis Fiberoptics Inc., Warren, NJ, USA) with a core diameter of 120 µm, a cladding thickness of 20 µm and an overcladding coating that leads to a total diameter of 490 µm for the fiber. For the DMA tests, the fiber is positioned in a DMA 8000 (Perkin Elmer, Waltham, MA, USA) with the single cantilever configuration, in this case the fiber is placed horizontally in the equipment with one end fixed, while the other is moved vertically with constant amplitude and frequency. The frequency interval ranges from 0.01 to ~5 Hz, which are logarithmically presented in an interval of three points per decade. Thus, the test was made in the frequencies of 0.01, 0.02, 0.05, 0.10, 0.21, 0.46, 1.00, 2.15 and 4.64 Hz. In this test, the fiber’s Young’s modulus is obtained with respect to the strain cycle frequency, where the test follows the ASTM D4065 standard for DMA procedures in polymers [32]. Figure 3 shows the POF fixation in the DMA employed in the Young’s modulus characterization, where the POF is positioned with one end at the fixed part and the other on the movable part, which oscillates periodically with controlled displacement and frequency.

The FBG inscription is made through the direct-write, plane-by-plane *fs* laser method with the modifications presented in [23,33]. The POF is positioned on a two-dimensional translation stage platform, with nanometer movement resolution and accuracy. The *fs* laser, operating at 517 nm with a pulse duration of 220 fs (HighQ laser femtoREGEN, Rothis, Austria), performs the direct grating inscription in the POF. A ×50 objective lens is employed in the beam focusing, which is carefully controlled to guarantee that the refractive index modifications are induced only in a part of the fiber core. In this way, the grating is inscribed in a limited depth of the fiber core (as confirmed by an optical microscope), where the spatial extent of the refractive index variation only occurs at the beam focus, since the CYTOP presents transparency and is not photosensitive in the 517 nm (*fs* laser operating wavelength). The inscribed POFBG has a physical length of ~1.2 mm. The inscription time is ~7 minutes, but with the advantageous feature of higher control of the gratings parameters, which is especially desirable for obtaining a single peak reflection spectrum in FBG inscription in multimode POFs. In addition, this higher controllability provided by the plane-by-plane method also leads to the grating inscription in a single attempt, which is not always the case with conventional grating inscription method.

**Figure 4:**
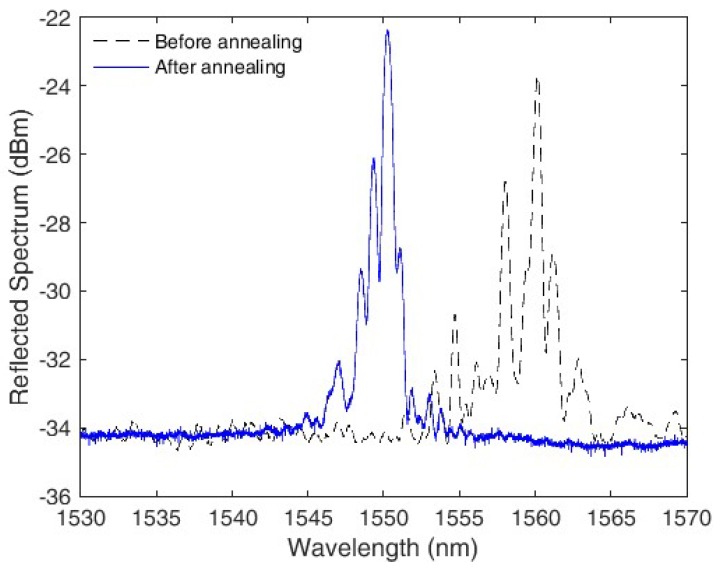
Polymer optical fiber Bragg gratings (POFBG) spectrum before and after the annealing.

Following the grating inscription, the sensor is subjected to annealing, which is a thermal treatment in which the POF is kept at temperatures close to its glass transition temperature for extended time periods of several hours [34]. The advantages of this thermal treatment is a reduction of the material Young’s modulus and the residual stress, the latter is an undesirable effect resulting from the fiber manufacturing process [35]. In this case, the annealing is made under water, since the high humidity can further increase its effectiveness [36]. In addition, the thermal treatment is made at a constant temperature of 90 °C for 24 h. Figure 4 presents the POFBG spectrum before and after the annealing. 

**Figure 5:**
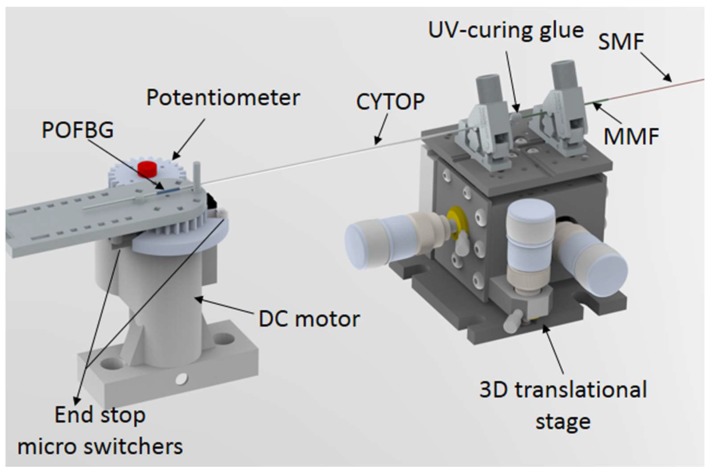
Experimental setup for the POFBG curvature sensor characterization.

Another essential step in the POFBG sensors’ development is their connectorizations. A less abrupt variation in the core diameter leads to lower optical power losses in the POF connectorization. For this reason, the CYTOP fiber is connectorized with a multimode silica fiber (MMF) though UV-curing glue applied after providing a good alignment between both fibers. Then, the MMF is fusion spliced in a single mode silica fiber (SMF), which is connected to the FBG interrogator (see Figure 5). The average loss due to the POF connectorization is ~3 dB.

The developed POFBG sensor is positioned on the experimental setup showed in Figure 5. The setup comprises of a DC motor with angular position and velocity control, where a potentiometer is connected with the DC motor and a gear (1:1 ratio) to provide the reference angle. The tests are performed over an angular range of 0 to 60°, which is within the knee joint angle interval during gait motion when walking [9]. In addition, two end-stop microswitches are employed to prevent system angles exceeding 60°. The CYTOP and MMF are fixed in the 3D translational stage, for the purpose of providing higher stability for the UV-joint during the oscillatory movements. The movement frequencies that are employed are 0.50, 0.61, 1.10, 1.32, 1.87 and 4.49 Hz. These frequencies were selected as they are close to the ones used in the material Young’s modulus characterization with respect to the movement frequency and are within the frequency range of the human lower limb movement.

## 4. Results and Discussion

### 4.1. Sensor Characterization

**Figure 6:**
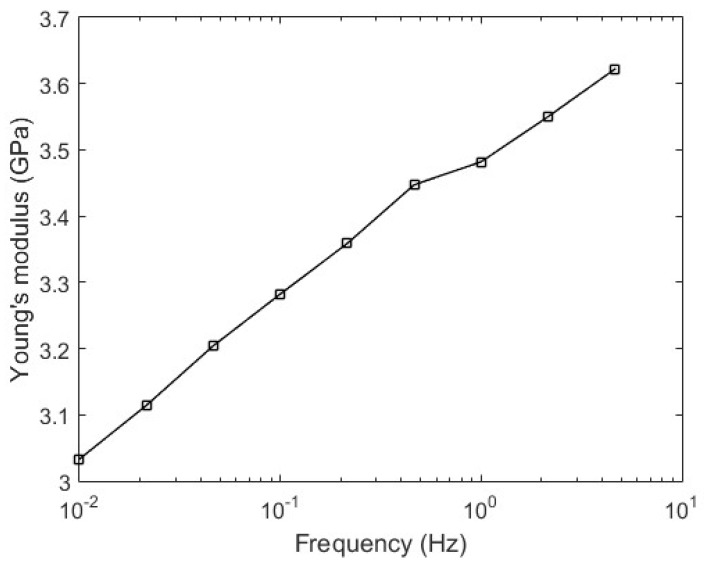
Cyclic transparent amorphous fluoropolymers (CYTOP) Young’s modulus as a function of the strain cycle frequency.

The first test is the fiber material characterization with respect to the strain cycle frequency. Figure 6 shows the obtained results, where there is an increase of the POF Young’s modulus with the increase in frequency. Thus, the angular velocity increase leads to a reduction of the sensor sensitivity (see Equation (4)).

**Figure 7:**
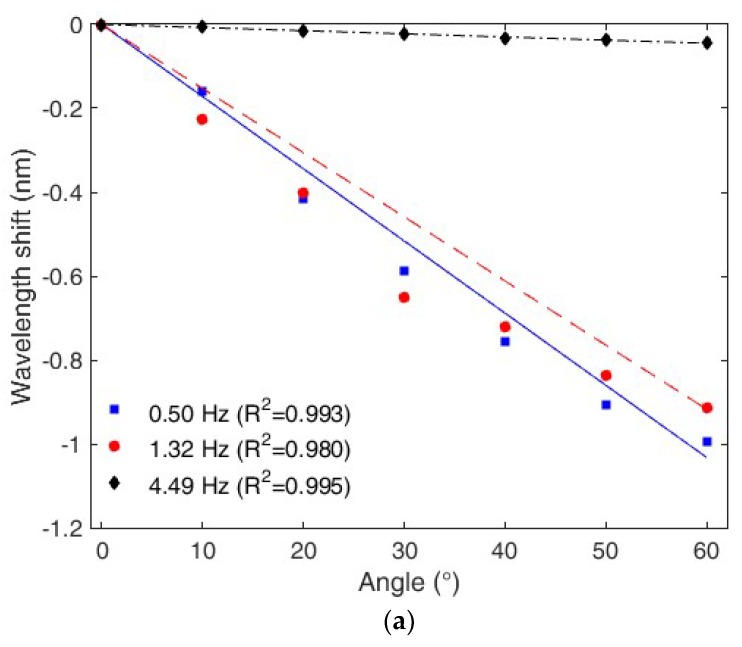

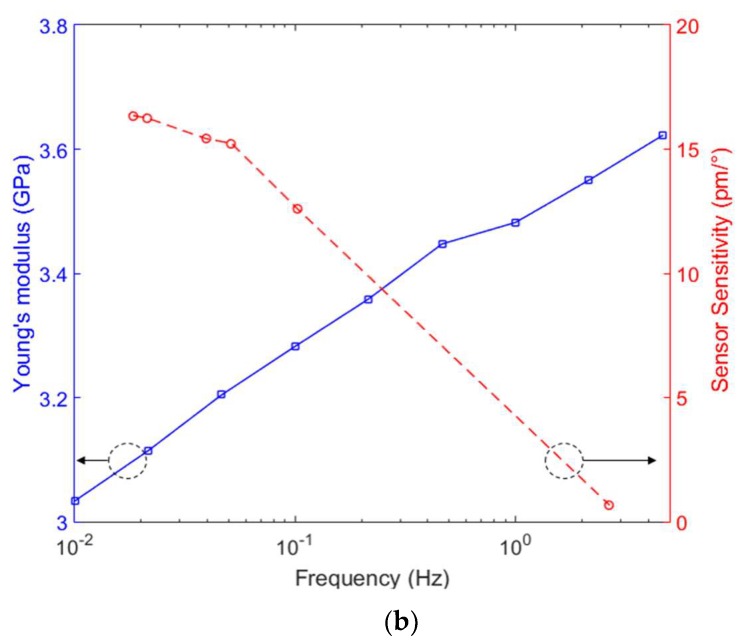
(**a**) POFBG curvature sensor response for three different frequencies (0.50, 1.32 and 4.49 Hz). (**b**) Comparison between the sensor sensitivity and Young’s modulus variations with respect to frequency.

In order to verify the sensitivity reduction with respect to the frequency increase, flexion cycles were performed for different frequencies. Figure 7a shows the response for three different frequencies, 0.50, 1.32 and 4.49 Hz. Although the sensor presents a low linearity at 1.32 Hz, the reason to employ a linear regression in this response is to show the sensor linearity, which is a performance parameter of sensor, obtained from the determination coefficient (*R*^2^) with respect to the linear fit [37]. Additionally, it is the same regression type as the one made for the other frequencies in which the *R*^2^ was higher than 0.99, which shows a linear behavior of the sensor. In addition, the direct relation between the sensor sensitivity and the Young’s modulus variation with frequency is shown in Figure 7b, where the same linear behavior was obtained for both material and sensor responses. It is worth to mention that the sensor presented similar repeatability in all performed tests (three cycles at each frequency), where the standard deviation was about 0.05 nm. For this reason, the effect of frequency variation on the sensor behavior is only related to the sensitivity and hysteresis variation in this case.

The linear regression between the wavelength shift (in nm) and the angle (in °) is presented in Equation (5) for the test at 0.5 Hz. This test was chosen for the regression due to its higher sensitivity. Equation (5) will be referred to as the uncompensated response, since it is the response without the application of any compensation technique.

(5) Δλ=−0.017α.

In addition, the responses presented in Figure 7a also shows the linear behavior of the sensor at each frequency. Therefore, the model “m3” is a linear regression relating the wavelength shift with sensitivity and hysteresis compensation and the angle.

Since the sensor displays a linear response to different frequencies, the model “m1” (see Figure 2) can be obtained by determining the linear regression between the sensor sensitivity and the frequency. Equation (6) presents the coefficients of this linear regression, where the value −0.004 is the rate of sensitivity variation with respect to the frequency and 0.019 as the sensor sensitivity (in pm/°) obtained in the lowest frequency tested.

(6) $$y_{s}=-0.004f+0.019.$$

Thus, the sensor sensitivity can be estimated at each frequency by applying Equation (6).

**Figure 8:**
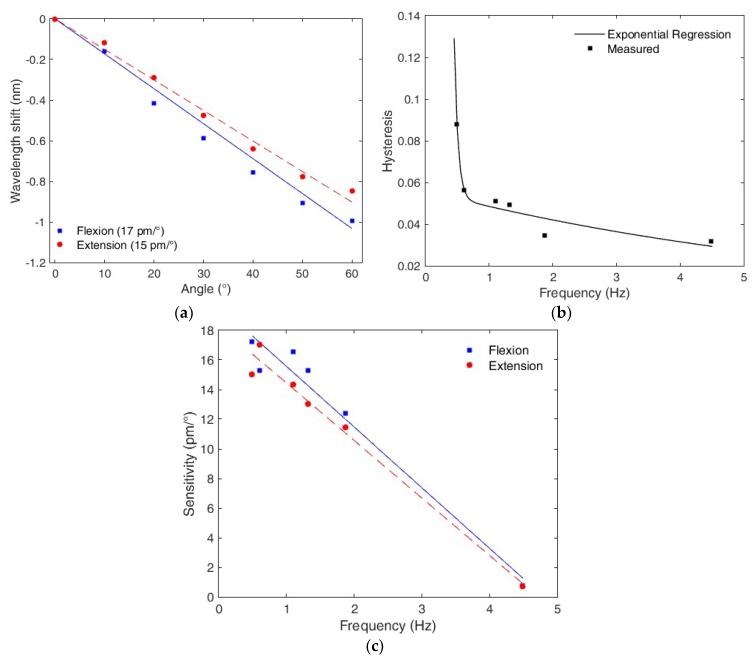
(**a**) Flexion and extension cycles at 0.5 Hz with the sensitivity of each cycle. (**b**) Hysteresis variation as a function of the frequency. (**c**) Sensitivity difference between flexion and extension cycles at each frequency.

A similar analysis is performed for the hysteresis compensation. In this case, the extension cycles are also analyzed, and the hysteresis is calculated as the offset between the flexion and extension curves. Figure 8a shows the sensor response for 0.5 Hz, whereas Figure 8b presents the hysteresis with respect to frequency. The relation between the sensor hysteresis and the oscillatory movement frequency follows a double-exponential regression, which is the same regression found for PMMA POF characterization through creep/stress relaxation tests [29] and in hysteresis compensation based on the material features [38]. These results indicate the influence of the creep and stress relaxation on the sensor response, which are also inherent effects of viscoelastic materials [27]. Hence, Equation (7) may be used to estimate the sensor hysteresis (*h*) (in nm/nm) based on its movement frequency (in Hz).

(7) h=168.20exp(−17.1f)+0.06exp(−0.14f)

Additionally, as presented in Figure 8a, there is also a sensitivity variation between flexion and extension cycles, which is a source of hysteresis. Therefore, for the hysteresis compensation, it is necessary not only compensate the offset between the curves, but also the sensitivity variation. For the tested frequencies, the sensitivity of the flexion cycle is greater than the extension cycle. Figure 8c depicts the sensitivity difference between flexion and extension cycles at each frequency (0.50, 0.61, 1.10, 1.32, 1.87 and 4.49 Hz), and we observe a hysteresis-related, lower sensitivity for the sensor extension cycles compared to the flexion cycles.

Equation (8) presents the sensitivity (in pm/°) with respect to frequency (in Hz) for the extension cycles recovered from Figure 8c.

(8) $$y_{s2}=-0.0038f-0.0197.$$

There will be two equations for hysteresis compensation, one for flexion and the other for extension. The flexion and extension movements can be detected by the direction of the angular velocity obtained after the derivative block in Figure 2. The equation for extension is comprised of the sensitivity correction with respect to frequency (Equation (8)) and the offset correction as a function of the frequency, detailed in Equation (7). The compensated equations for flexion and extension cycles are given by Equations (9) and (10), respectively. It is worth to mention that Equation (10) presents the influence of the hysteresis on the sensor response. However, the hysteresis variation as a function of the frequency was already characterized in Equation (7). Thus, by substituting Equation (7) in (10), it is possible to obtain an equation that depends only on *α* and *f*, which is similar to Equation (9).

(9) Δλ=(−0.004f+0.019)α.

(10) Δλ=(−0.0038f−0.0197+h)α.

### 4.2. Compensation Technique Verification

**Figure 9:**
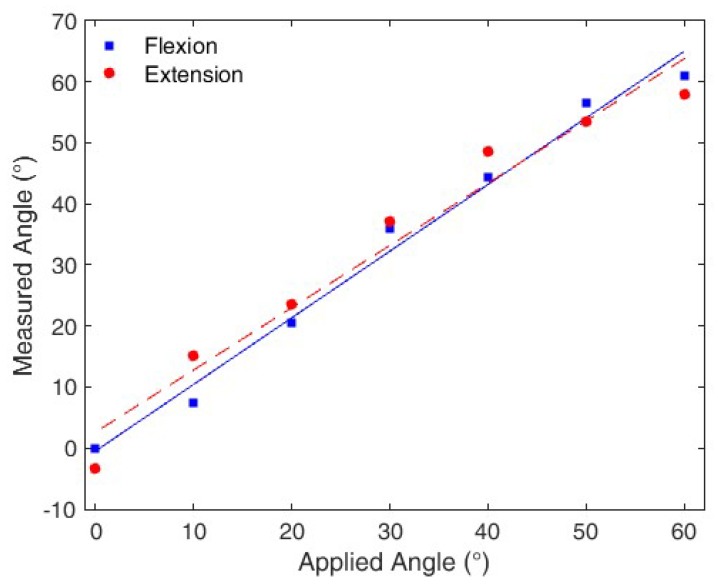
Measured angles for flexion and extension cycles after applying the compensation technique.

The compensation technique presented in the previous sections is applied and verified, where Equation (9) is applied for the flexion movement and Equation (10) for extension. Figure 9 shows results of the compensated response for the test at 1.10 Hz, for which we found the highest hysteresis reduction.

**Figure 10:**
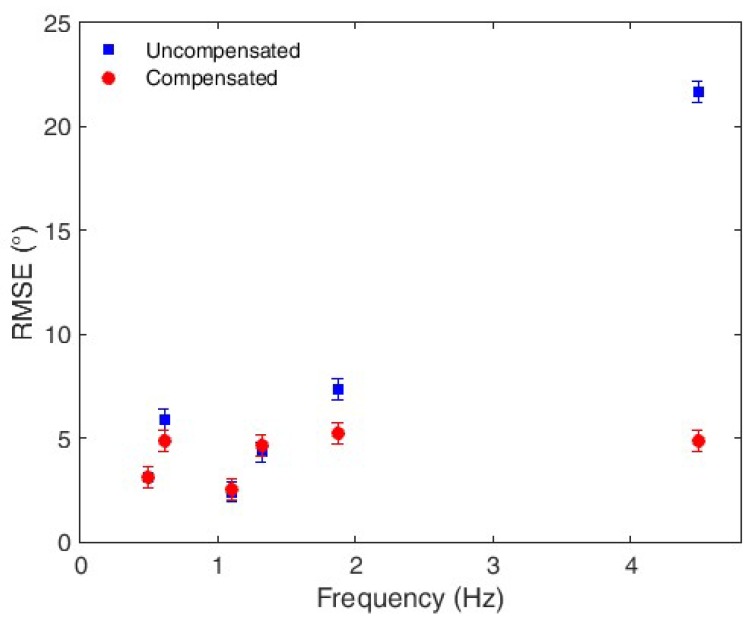
RMSE of the compensated and uncompensated responses at each tested frequency.

The comparison between the compensated and uncompensated responses is made with respect to the sensors’ hysteresis and root mean squared error (RMSE). The uncompensated response is obtained by substituting the wavelength shift in Equation (5) that results in the estimated angle, whereas the compensated response is obtained by applying Equations (9) and (10). The compensation technique verification occurs in two steps; first, validation of the frequency cross-sensitivity compensation, followed by the hysteresis compensation validation. For the cross-sensitivity compensation analysis, the RMSE between the applied and measured angles is compared at each frequency for the compensated and uncompensated cases. Figure 10 shows the results obtained, where the RMSE is presented with respect to the frequency. As expected, the results at 0.5 Hz show the same RMSE, given that Equation (5) (employed for the uncompensated response analysis) is obtained at the 0.5-Hz flexion cycle. As the frequency increases, there is a sharp increase in the error of the uncompensated response, whereas compensated responses are almost constant. In addition, we note that the errors observed in the compensated measurements are related to sensor nonlinearities that lead to a reduction of the correlation coefficient between the sensor response and the linear regression. Nevertheless, the correlation coefficient is higher than 0.98 in all tests. The compensated and uncompensated responses presented similar RMSEs for all frequencies below 1.5 Hz. The difference between the responses increases when the frequency exceeds 1.5 Hz, as the sensitivity exhibits higher variations at higher frequencies. The mean RMSE of the compensated response is 4.20°, whereas that of the uncompensated response is 7.45°. Therefore, the compensation technique leads to a comparative RMSE reduction of 44%.

**Figure 11:**
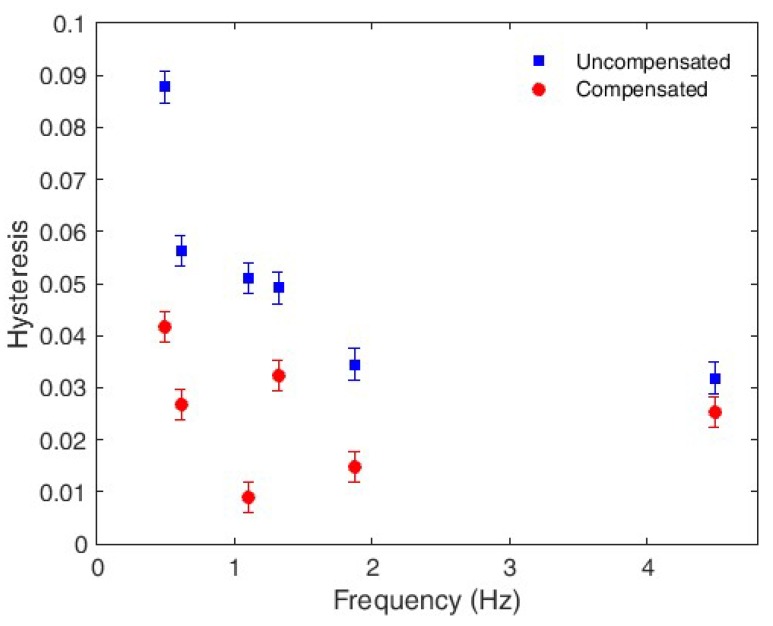
Hysteresis of the compensated and uncompensated responses at each tested frequency.

Verification of the second step of the compensation technique requires a comparison of the sensor hysteresis before and after the compensation. Once again, the results are presented as a function of the movement frequency. In this case, the uncompensated hysteresis showed a downward trend with increasing frequency. Although the hysteresis was reduced for all tested frequencies, it less effective for frequencies >1.5 Hz. Nevertheless, there is an improvement that is observed across the frequency range (see Figure 11).

The mean hysteresis before the application of the compensation technique is 0.0403, and 0.0220 following compensation; a reduction of 55%. Thus, proposed compensation technique for movement frequency cross-sensitivity and hysteresis mitigation is validated, where the frequency cross-sensitivity of the sensor is compensated through the sensor characterization at different frequencies and with the material characterization through the DMA. Furthermore, the hysteresis is compensated through the sensor characterization under different oscillatory movement frequency conditions. In these tests, the sensor hysteresis shows an exponential behavior as a function of the movement frequency similar to the one found in the creep response characterization of POFs, as anticipated [29].

## 5. Conclusions

In this paper, a POFBG curvature sensor is proposed with the POFBG inscribed in CYTOP fibers using a *fs* laser. A compensation technique for movement frequency cross-sensitivity and hysteresis mitigation is proposed and validated for flexion and extension cycles at different frequencies. The proposed compensation technique leads to a reduction of both RMSE and hysteresis, where the mean reduction was 44% and 55% of the RMSE and hysteresis, respectively.

The technique presented in this paper is a calibration methodology for POFBG curvature sensors that can be applied to POFBGs of different materials or for different frequency intervals. Since polymers are also sensitive to temperature variations [30], an analogous technique can be employed in the POFBG curvature sensor for the compensation of the temperature effects on the sensor response, as will be explored in future investigations that also include real applications of the proposed curvature sensor. Another future investigation is the POFBG analysis at higher frequencies and lower angular displacements for vibration measurements.

## Figures and Tables

**Table 1:** Parameters employed in the sensor simulation.

Symbol	Parameter	Value
*P_e_*	Photoelastic constant	0.30
*λ_B_*	Bragg wavelength	1550.00 nm
*υ*	Poisson’s ratio	0.42
*E*_1_	Young’s modulus at f1	3.00 GPa
*E*_2_	Young’s modulus at f2	2.00 GPa
*I*	Moment of inertia	4.53 × 10^−14^ m^4^
*M*	Bending constant	100 Nm
*h*1	Hysteresis at f1	0.10
*h*2	Hysteresis at f2	−0.05
